# Sip1, an AP-1 Accessory Protein in Fission Yeast, Is Required for Localization of Rho3 GTPase

**DOI:** 10.1371/journal.pone.0068488

**Published:** 2013-07-01

**Authors:** Yang Yu, Cuifang Li, Ayako Kita, Yuta Katayama, Koji Kubouchi, Masako Udo, Yukako Imanaka, Shiho Ueda, Takashi Masuko, Reiko Sugiura

**Affiliations:** 1 Laboratory of Molecular Pharmacogenomics, School of Pharmaceutical Sciences, Kinki University, Kowakae, Higashi-Osaka, Japan; 2 Japan Society for the Promotion of Science, Tokyo, Japan; 3 Laboratory of Molecular Cell Biology, School of Pharmaceutical Sciences, Kinki University, Kowakae, Higashi-Osaka, Japan; Institute of Molecular and Cell Biology, Biopolis, United States of America

## Abstract

Rho family GTPases act as molecular switches to regulate a range of physiological functions, including the regulation of the actin-based cytoskeleton, membrane trafficking, cell morphology, nuclear gene expression, and cell growth. Rho function is regulated by its ability to bind GTP and by its localization. We previously demonstrated functional and physical interactions between Rho3 and the clathrin-associated adaptor protein-1 (AP-1) complex, which revealed a role of Rho3 in regulating Golgi/endosomal trafficking in fission yeast. Sip1, a conserved AP-1 accessory protein, recruits the AP-1 complex to the Golgi/endosomes through physical interaction. In this study, we showed that Sip1 is required for Rho3 localization. First, overexpression of *rho3* ^+^ suppressed defective membrane trafficking associated with *sip1-i4* mutant cells, including defects in vacuolar fusion, Golgi/endosomal trafficking and secretion. Notably, Sip1 interacted with Rho3, and GFP-Rho3, similar to Apm1-GFP, did not properly localize to the Golgi/endosomes in *sip1-i4* mutant cells at 27°C. Interestingly, the C-terminal region of Sip1 is required for its localization to the Golgi/endosomes, because *Sip1-i4*-GFP protein failed to properly localize to Golgi/endosomes, whereas the fluorescence of Sip1ΔN mutant protein co-localized with that of FM4-64. Consistently, in the *sip1-i4* mutant cells, which lack the C-terminal region of Sip1, binding between Apm1 and Rho3 was greatly impaired, presumably due to mislocalization of these proteins in the *sip1-i4* mutant cells. Furthermore, the interaction between Apm1 and Rho3 as well as Rho3 localization to the Golgi/endosomes were significantly rescued in *sip1-i4* mutant cells by the expression of Sip1ΔN. Taken together, these results suggest that Sip1 recruits Rho3 to the Golgi/endosomes through physical interaction and enhances the formation of the Golgi/endosome AP-1/Rho3 complex, thereby promoting crosstalk between AP-1 and Rho3 in the regulation of Golgi/endosomal trafficking in fission yeast.

## Introduction

In eukaryotic cells, Rho family small GTPases play a crucial role in numerous important cellular functions, including polarized growth through reorganization of the actin cytoskeleton, regulation of secretory vesicle transport, and gene transcription [1,2]. Most Rho proteins act as switches by cycling between active (GTP-bound) and inactive (GDP-bound) conformations [3]. Guanine nucleotide exchange factors (GEFs) promote the exchange of GTP for GDP. GTPase-activating proteins (GAPs) enhance intrinsic GTP-hydrolysis activity, leading to GTPase inactivation. Guanine-nucleotide -dissociation inhibitors (GDIs) bind to prenylated GDP-bound Rho proteins and allow translocation between membranes and the cytosol [1,3]. Most small G proteins are localized either in the cytosol or on membranes, and each small G protein is localized to a specific membrane [1]. This localization is mediated by posttranslational modifications with lipid; the mechanism involves prenylation of small G proteins [4], and this modification is necessary for proper localization as well as function of small G proteins [5]. Thus, the mechanism(s) that regulate the intracellular location and localized activation of Rho GTPases, including prenylation, form another important means by which the Rho family is regulated. Although detailed information is available on numerous Rho target proteins that mediate Rho signaling, Rho-interacting proteins that affect Rho-dependent signaling processes through spatial control are relatively unknown. The budding yeast *Saccharomyces cerevisiae* and the fission yeast *Schizosaccharomyces pombe* have 6 Rho GTPases, named Rho1-5 and Cdc42 [6]. Because of their simplicity and straight forward genetics, both these yeasts are excellent models for studying the basic mechanisms of Rho regulation and Rho-dependent signaling processes [6]. Rho3 is a GTPase that plays important roles in membrane trafficking and polarized growth in both these yeasts [6]. In budding yeast, Rho3 regulates polarized secretion and the actin cytoskeleton by interacting with the Exo70 component of the exocyst and Myo2 [7]. In the fission yeast *Schizosaccharomyces pombe*, Rho3 is implicated in polarized cell growth through both Formin and by regulating the proper localization of the exocyst and secretion [8,9]. Recent studies including ours, revealed a novel role of Rho3 in the Golgi/endosomal trafficking, partly through physical and/or functional interaction with the clathrin-associated adaptor protein-1 (AP-1) complex and Cdc42 in fission yeast [9,10]. We also showed that the AP-1 complex mutant strains showed defects in Golgi/endosomal trafficking, secretion and vacuole fusion [11,12] and that Sip1, the AP-1 accessory recruits the AP-1 complex to the Golgi/endosomes by identifying the *sip1-i4* mutant allele, which abolished the endosomal localization of the AP-1 complex [13].

Sip1 is a homolog of Laa1 in the budding yeast [14] and p200 in higher eukaryotes [15], both of which belong to the emerging family of AP-1 interacting partners. To understand the molecular function of the AP-1 accessory protein and elucidate the pathways interacting with Sip1/AP-1-mediated trafficking, we screened for the multi-copy suppressor of the temperature-sensitive growth of *sip1-i4* cells and identified the *rho3* ^+^ gene. In the present study, we investigated the role of Rho3 in the Sip1/AP-1-mediated Golgi/endosomal membrane trafficking pathway. We found that Sip1, an AP-1 accessory protein, also serves as a binding partner for Rho3, thereby regulating its intracellular localization in the Golgi/endosomes. In *sip1-i4* mutant cells, the formation of the Rho3/AP-1 complex was impaired. Thus, we propose a role for this AP-1 accessory protein to recruit the small GTPase Rho3 to its proper cellular localization and facilitate its interaction with AP-1 complex. 

## Materials and Methods

### Strains, Media and Genetic and Molecular Biology Methods

*Schizosaccharomyces pombe* strains used in this study are listed in Table 1. The complete and minimal media used were yeast extract-peptone-dextrose (YPD) and Edinburgh minimal medium (EMM), respectively. Standard genetic and recombinant DNA methods [16] were used unless otherwise stated. FK506 was provided by Astellas Pharma, Inc. (Tokyo, Japan). Genomic DNA clones were provided by the National Bio Resource Project, Yeast Genetic Resource Center (Graduate School of Science, Osaka City University).

**Table 1:** *Schizosaccharomyces pombe* strains used in this study.

**Strain**	**Genotype**	**Reference**
HM123	*h* ^*-*^ * leu1-32*	Our stock
KP456	*h* ^*-*^ * leu1-32 ura4-D18*	Our stock
SP733	*h* ^*-*^ * leu1-32 sip1-i4*	Yu et al. (2012)
KP630	*h- leu1-32 ura4-D18 apm1::ura4* ^*+*^	Kita et al. (2004)
SP736	*h- leu1-32 ura4-D18 sip1-i4*	Yu et al. (2012)
KP1754	*h- leu1-32 ura4-D18 nmt1 GST-sip1+::KanMx6*	Our stock
KP1375	*h- leu1-32 ura4-D18 rho3::ura4* ^*+*^	Our stock
KP1915	*h- leu1-32 ura4-D18 GFP-sip1* ^*+*^ *::KanMx6*	Our stock
SP936	*h- leu1-32 ura4-D18 rho3::ura4* ^*+*^ * sip1* ^*+*^ *-GFP::KanMx6*	This study
SP1730	*h- leu1-32 ura4-D18 GFP-sip1* ^*+*^ *::KanMx6 anp1+-linker-mCherry::ura4* ^*+*^	Yu et al. (2012)
SP1966	*h- or+ leu1-32 ura4-D18 GFP-sip1+::KanMx6 sec 72* ^*+*^ *-mCherry::ura4* ^*+*^	Yu et al. (2012)
SP2080	*h- leu1-32 ura4-D18 GFP-sip1* ^*+*^ *::KanMx6 anp1+-linker-mCherry::ura4* ^*+*^ * rho3: :KanMx6*	This study
SP2082	*h- or+ leu1-32 ura4-D18 GFP-sip1* ^*+*^ *::KanMx6 sec 72* ^*+*^ *-mCherry::ura4+ rho3::ura4* ^*+*^	This study

### Cloning of the *rho3* ^+^ genes

The *sip1-i4* mutant was transformed using an *S. pombe* genomic DNA library constructed in the vector pDB248. Leu+ transformants were replica-plated onto YPD plates at 36°C, and the plasmid DNA was recovered from transformants that exhibited plasmid-dependent rescue. The plasmids that complemented the temperature sensitivity of the *sip1-i4* mutant were cloned and sequenced. The suppressing plasmids fell into 2 classes: 1 containing *sip1* ^*+*^ and the other containing *rho3* ^*+*^ (SPAC23C4.08).

### Plasmid Construction

The *sip1-i4* mutation gene (*sip1-i4*) was amplified with Vent DNA polymerase by polymerase chain reaction (PCR) using the genomic DNA of wild-type (wt) cells as a template. The sense and antisense primers were 5′-GAA GAT CTT ATG TCG TTA GCA TCA TTG CCG CTC G-3′, and 5′-GAA GAT CTG CGG CCG CCT AAA GTA GCA ATA CGA AG-3′, respectively. The amplified product containing *sip1* was subcloned into BglII/NotI sites of BlueScriptSK (+) (Stratagene). The amino-terminal truncation of Sip1 (Sip1∆N) was amplified with Vent DNA polymerase by PCR using the genomic DNA of wt cells as a template. The sense and antisense primers were 5′-CGG GAT CCC ATG ATC AGC TCT GCT TTT AGT TCC-3′, and 5′-CGG GAT CCG CGG CCG CCC TCA ACA TTT TGT ATT AAG-3′, respectively. The amplified product containing Sip1∆N was subcloned into *Bam*HI sites of BlueScriptSK (+) (Stratagene). The thiamine-repressible *nmt1* promoter was used for ectopic protein expression [17]. Expression was repressed by the addition of 4 µM thiamine to EMM. To assess subcellular localization, *Sip1*-*i4* mutant protein (Sip1∆C) and Sip1∆N proteins were tagged at their C termini with green fluorescent protein (GFP) carrying the S65T mutation [18]. Similarly, Sip1∆C and Sip1∆N proteins were tagged at their C termini with glutathione-S-transferase (GST). These constructs were confirmed by restriction digestion and sequence analysis. The functionality of the obtained proteins was verified by complementation of the *sip1-i4* mutant cells.

### Protein Expression and Site-Directed Mutagenesis

The thiamine-repressible *nmt1* promoter was used for protein expression in yeast [17]. Protein expression was repressed by the addition of 4 µg/ml thiamine to EMM and was induced by washing and incubating the cells in EMM without thiamine. The GST- or GFP-fused gene was subcloned into the pREP1 vector to obtain maximum expression of the fused gene using pREP1 of the *nmt1* promoter. The site-directed mutagenesis was performed using the Quick Change Site-Directed Mutagenesis Kit (Stratagene).

### Microscopy and Miscellaneous Methods

Light microscopy methods (e.g., fluorescence microscopy) were performed as described previously [12]. Photographs were taken using AxioImager A1 (Carl Zeiss, Germany) equipped with an AxioCam MRm camera (Carl Zeiss, Germany) and AxioVision software (Carl Zeiss). Images were processed with the CorelDRAW software (Corel, Ottawa, Ontario, Canada). Furthermore, FM4-64 labeling, the localization of GFP-Syb1, and measurements of acid phosphatase secretion were performed as described previously [12].

### Image Quantification

All image quantification analyses were performed for 3 individual datasets, which summed up to 150 counted cells.

### Rho3 Antibodies

Monoclonal antibodies against Rho3 were raised by using purified Rho3 from *S. pombe*. For the first immunization, female F344/N rats at seven weeks of age (Shimizu Animal Farm, Kyoto, Japan) were housed in a controlled environment at 22°C in a specific-pathogen-free facility, and were administered an intraperitoneal injection of recombinant GST-fused Rho3 protein from *S. pombe* (GST-Rho3; 100 µg in 500 µl of saline in each rat) emulsified with an equal volume of complete Freund’s adjuvant (Difco, Detroit, MI) followed by a booster intraperitoneal injection of GST-Rho3 (100 µg in 500 ml of saline) without adjuvant at a 10-day interval. Four days later, rats were sacrificed, antisera were collected, and the immune spleen cells (1.0 ×10^8^) were fused with P3×63Ag8.653 mouse myeloma cells (2.5 × 10^7^) using 50% polyethylene glycol 1540 (Roche, Penzberg, Germany). After the cell fusion, hybridoma cells were selected in 7% FBS-containing RPMI 1640 medium (Sigma-Aldrich, St. Louis, MO) supplemented with hypoxanthine, aminopterin and thymidine (50 × HAT; Invitrogen, Carlsbad, CA). Nine to twelve days later, hybridoma antibodies in the culture medium were assessed for positive reaction to GST-Rho3 and negative reaction to GST by enzyme-linked immunosorbent assay (ELISA). Rats were used with the approval of the Committee for the Care and Use of Laboratory Animals at Kinki University.

### Immunostaining of Whole Cells

Cells were cultured in YES medium for 20h, and then fixed by adding methanol on -80 °C. After fixed the cells were washed 3 times with PEM (100 mM PIPES 1 mM EGTA, 1 mM MgCl_2_, pH 6.9). Cells were treated with PEMS (PEM + 1.2 M sorbitol) containing zymolyase 20T (0.5 mg per mL) at 37°C until approximately 10% of the cells lost their cell walls as observed under a microscope. Subsequently, the cells were washed with PEM 3 times and were incubated for 2 h at room temperature with 100 µL of 1% PEMBAL (PEM + 1% BSA. 0.1% sodium azide, 1% L-Lysine hydrochloride) containing anti-Rho3 antibodies. After incubation, the cells were washed 3 times with PEMBAL and treated with 1:100-diluted FITC-conjugated goat anti-rat immunoglobulin (Jackson Research Laboratories) in 50 µL of PEMBAL in the dark for 2 h at room temperature. The cells were washed 3 times with PEMBAL and mounted on slides in PBS for observation by fluorescence microscopy.

## Results

### Identification of *rho3* ^+^ as a multicopy suppressor of *sip1-i4* mutants

**Figure 1:**
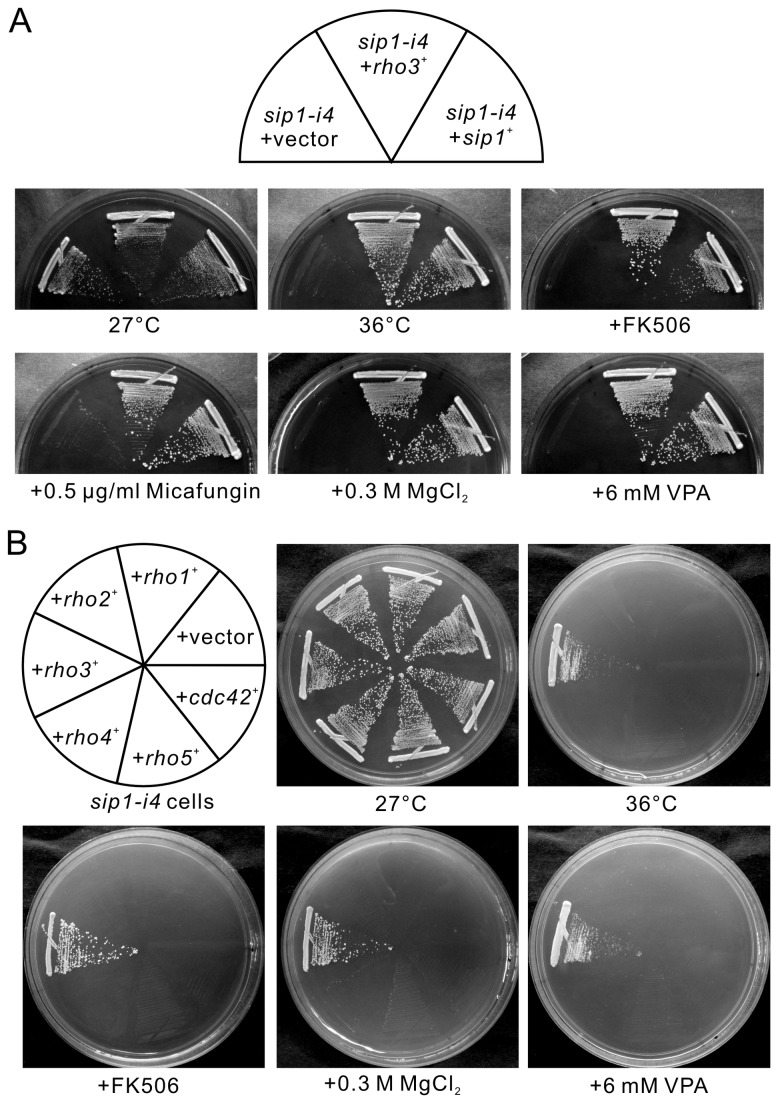
Isolation of Rho3 as a multicopy suppressor of *sip1-i4* mutant cells. (A) *sip1-i4* cells were transformed with either the pDB248 multicopy vector, vector containing *sip1* ^*+*^ or vector containing *rho3* ^*+*^. Cells were then streaked onto plates containing 0.5 µg/mL FK506, 0.5 µg/mL micafungin, 0.3 M MgCl_2_, or 6 mM valproic acid and incubated at 27°C for 4 d or at 36°C for 3 d, respectively. (B) *sip1-i4* cells transformed with the multicopy vector pDB248, or the genome DNA clones containing *rho1* ^*+*^, *rho2* ^*+*^, *rho3* ^*+*^, *rho4* ^*+*^, *rho5* ^*+*^, or *cdc42* ^*+*^ were streaked onto plates containing 0.5 µg/mL FK506, 0.3 M MgCl_2_, or 6 mM valproic acid then incubated at 27°C for 4 d or at 36°C for 3 d, respectively.

In a previous report, we showed that the *sip1-i4* mutant strain was thermosensitive (Figure 1A, *sip1-i4* + vector) [13]. To identify novel genes involved in Sip1 function or Sip1-mediated membrane trafficking, we screened a fission yeast genomic library to isolate genes that when overexpressed, could suppress the temperature sensitivity of the *sip1-i4* mutant cells. One of these genes was *rho3* ^*+*^, which encodes a member of the Rho family of small GTPases. As shown in Figure 1A, overexpression of *rho3* ^*+*^ suppressed the temperature-sensitive growth of the *sip1-i4* mutants (Figure 1A; *sip1-i4* +*rho3* ^+^, 36°C). The *rho3* ^*+*^ gene overexpression also suppressed phenotypes associated with the *sip1-i4* mutants, including the sensitivity to FK506, a specific inhibitor of calcineurin phosphatase, MgCl_2_, the cell wall–damaging agent micafungin, and valproic acid (VPA; Figure 1A) [13].

To determine the specificity of Rho3 in the suppression of the *sip1-i4* mutants, we investigated the effects of other genes that encode members of the Rho family of small GTPases present in the fission yeast genome. To test the suppression capabilities of all the 6 fission yeast Rho family members, the *sip1-i4* mutant cells transformed with *rho1* ^*+*^, *rho2* ^*+*^, *rho3* ^*+*^, *rho4* ^*+*^, *rho5* ^*+*^, or *cdc42* ^+^ were tested for growth at 36°C or in media containing FK506, 0.3 M MgCl_2_, and 6 mM VPA. Rho3 overexpression, but not that of the other Rho family members, could suppress the sensitivities of *sip1-i4* mutant cells to temperature (36°C), the immunosuppressive drug FK506, MgCl_2_, and VPA (Figure 1B). These results clearly indicated that Rho3 exhibits highly specific suppression of various sensitivities of *sip1-i4* mutant cells in all the members of the fission yeast Rho family.

### Rho3 suppresses defective membrane trafficking in *sip1-i4* mutant cells

**Figure 2:**
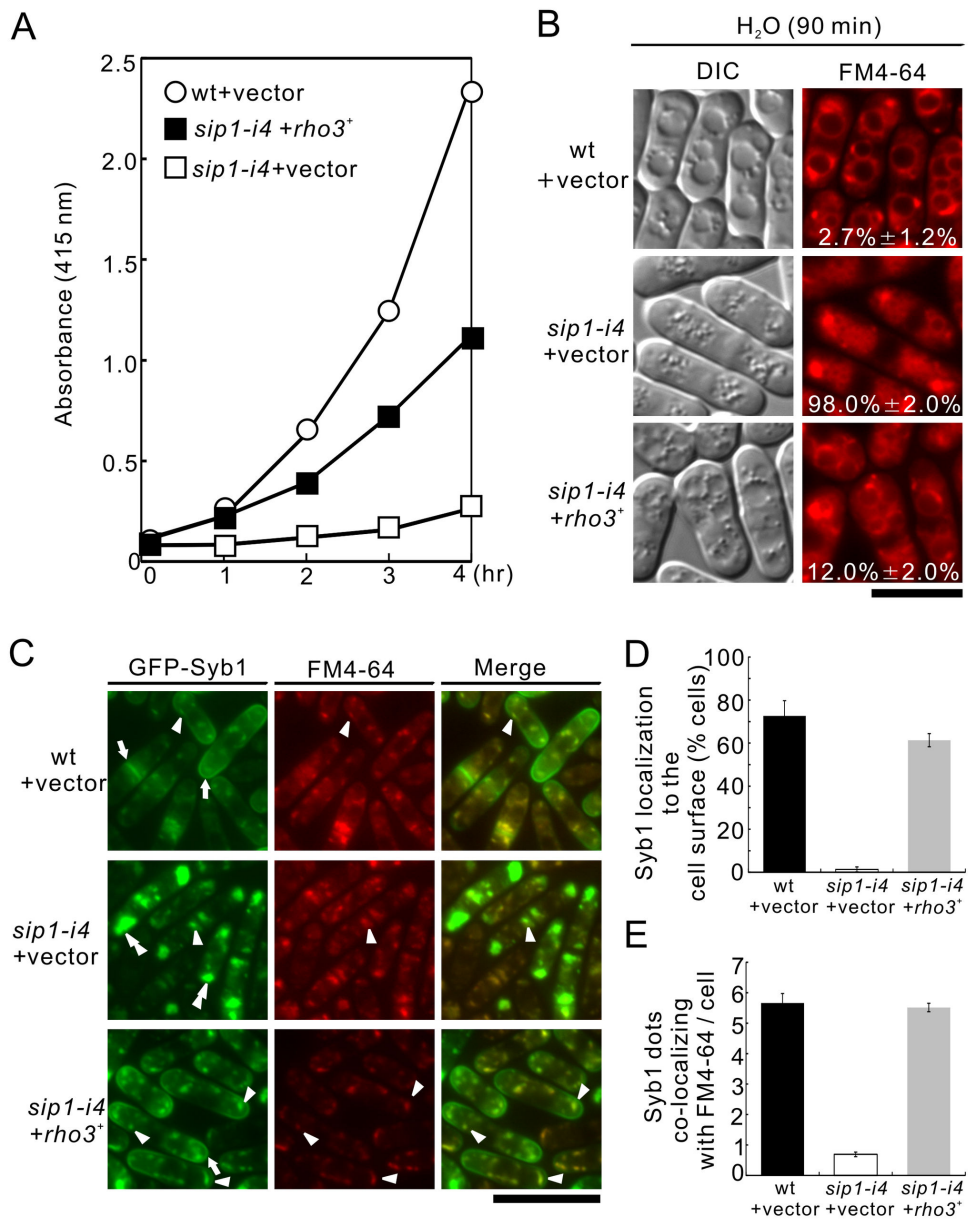
Rho3 suppresses various phenotypes associated with *sip1-i4* mutant cells. (A) Rho3 suppresses the defective secretion of acid phosphatase in *sip1-i4* mutant cells. Wild-type (wt) and *sip1-i4* cells, which were transformed with either the pDB248 vector or *rho3* ^+^-containing vector, were assayed for acid phosphatase activity. Data are representative of 3 independent experiments. (B) Rho3 suppresses the defects in vacuole fusion in *sip1-i4* cells. The wt and *sip1-i4* cells transformed with pDB248 or the vector containing *rho3* ^+^ were cultured in YPD medium at 27°C. Cells were harvested, labeled with FM4-64 fluorescent dye for 60 min, resuspended in water, and examined by fluorescence microscopy. Bar, 10 µm. The number in the image indicates the percentage of cells with fragmented vacuoles. Data from at least 3 independent experiments are expressed as means ± standard deviations. (C) Rho3 suppresses GFP-Syb1 mislocalization in *sip1-i4* mutant cells. The wt and *sip1-i4* cells expressing GFP-Syb1 transformed with pDB248 or the vector containing *rho3* ^+^ were cultured in YPD medium at 27°C. GFP-Syb1 localization was examined under a fluorescence microscope. Arrowheads indicate the dot-like structures of GFP-Syb1 and the Golgi/endosomes stained with FM4-64, double arrowheads indicate cytoplasmic accumulation, and arrows indicate the concentrated fluorescence at the medial region and cell surface. Bar, 10 µm. (D) Quantitative analysis of the number of Syb1 dots that co-localized with FM4-64/cell. (E) Percentage of cells in which Syb1 was localized at the cell surface. Cells in D and E were the same as those indicated in C.

We next investigated whether Rho3 overexpression can rescue the membrane-trafficking defects in *sip1-i4* mutant cells. Even at the permissive temperature (27°C), the *sip1-i4* cells secreted less acid -phosphatase than the wild-type cells (Figure 2A). Overexpression of *rho3* ^*+*^ partly but significantly restored acid –phosphatase secretion in the *sip1-i4* mutant cells (Figure 2A, *sip1*-*i4* + *rho3* ^+^). Following this, we examined the effect of Rho3 overexpression on vacuole fusion observed in the *sip1-i4* mutant cells. After the cells were labeled with FM4-64 for 60 min, harvested, washed, and resuspended in water for 90 min, the wild-type cells showed large prominent vacuoles resulting from vacuole fusion, (Figure 2B, wt + vector, 2.7% ± 1.2%). In contrast, the *sip1-i4* mutant cells showed numerous small vacuoles (Figure 2B, *sip1-i4* + vector, 98.0% ± 2.0%), indicating a defect in vacuole fusion. Notably, overexpression of the *rho3* ^*+*^ restored vacuole fusion in the *sip1-i4* mutant cells, because the *sip1-i4* mutant cells harboring Rho3 contained larger vacuoles (Figure 2B, *sip1-i4* + *rho3* ^+^, 12.0% ± 2.0%) than those harboring the vector alone. We also examined the effect of *rho3* ^*+*^ overexpression on defects in Golgi/endosomal membrane trafficking in the *sip1-i4* mutant cells. For this purpose, we used Syb1, the synaptobrevin in fission yeast. This is a vesicle-associated membrane protein that can be copurified with secretory vesicles [19]. GFP-Syb1 fluorescence in the wild-type cells was enriched in the medial region or cell ends (Figure 2C, wt + vector, arrows) and was detected in the Golgi/endosomes, as evidenced by its co-localization with FM4-64 (Figure 2C, wt + vector, arrowheads, Figure 2D). In contrast, GFP-Syb1 failed to localize on the cell surface (Figure 2C, *sip1-i4* + vector, arrows, 2E), or in the Golgi/endosomes in the *sip1-i4* mutant cells. Instead, GFP-Syb1 was observed as large, brightly fluorescent dots in the cytoplasm at 27°C (Figure 2C, *sip1-i4* + vector, double arrowheads, Figure 2D). Notably, GFP-Syb1 was visible at the cell ends in the *sip1-i4* mutant cells that harbored *rho3* ^+^ (Figure 2C, *sip1-i4 + rho3* ^+^, arrows, Figure 2E), and Rho3 overexpression recovered normal Syb1 dots that co-localized with FM4-64 (Figure 2C, *sip1-i4 + rho3* ^+^, arrowheads, Figure 2D). Thus, Rho3 restores Syb1 localization in *sip1-i4* cells.

### Sip1 interacts with Rho3 signaling

**Figure 3:**
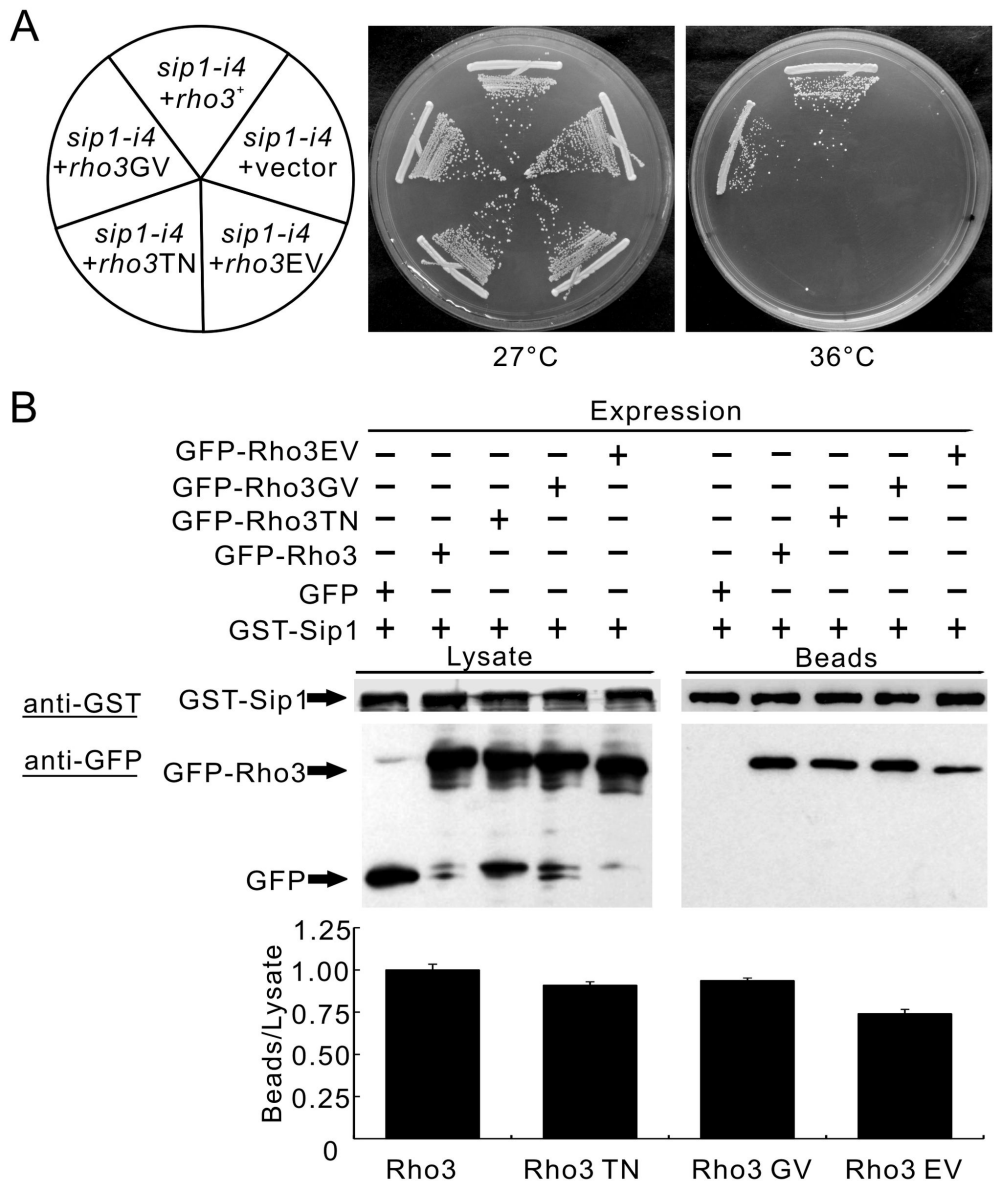
Functional and physical interactions between Rho3 and Sip1. (A) Rho3 suppresses *sip1-i4* mutant cells (*sip1-i4*) in a GTP- and effector domain-dependent manner. The *sip1-i4* cells were transformed with the pDB248 multi-copy vector or the vector containing *rho3* ^*+*^, *rho3*GV, *rho3*TN, and *rho3*EV expressed from its endogenous promoter. These cells were streaked onto YES plates and then incubated at 27°C for 4 d or at 36°C for 3 d, respectively. (B) Binding assay for Sip1 and Rho3. GST pull-down experiments were performed using chromosome-borne GST-Sip1 expressed under the control of the *nmt1* promoter. Cells expressing GFP alone, or GFP-Rho3, GFP-Rho3GV, GFP-Rho3TN, or GFP-Rho3EV were harvested and their lysates were incubated with the purified full-length Sip1 fused GST protein. GST-tagged Sip1 was precipitated with glutathione beads, washed extensively, subjected to SDS-PAGE, immunoblotted using anti-GFP or anti-GST antibodies and visualized by autoradiography. Lower panel: Quantitation of GFP-tagged various mutant forms of Rho3 beads protein levels by densitometry of the expressed bands against that of the lysate protein levels as shown in B. Data from at least three independent experiments are expressed as means ± standard deviations.

To investigate the functional relationship between Sip1 and Rho3 signaling, we examined the effects of various mutant forms of Rho3 on the temperature -sensitivity of the *sip1-i4* mutant cells. The following mutants were used: a GDP-locked variant of Rho3 in which the conserved (among Rho3 proteins) Thr27 was replaced with Asn (Rho3T27N), a GTP-locked variant of Rho3 (Rho3G22V) in which the conserved Gly22 was replaced with Val, and an effector domain mutant Rho3 (Rho3E48V) in which the conserved Glu48 was replaced with Val [10]. Similar to wild-type Rho3, overexpression of the dominant-active Rho3GV mutant suppressed the temperature-sensitive growth of the *sip1-i4* mutant cells (Figure 3A, *sip1-i4* + *rho3*GV). In contrast, both Rho3T27N and Rho3E48V overexpression failed to suppress the sensitivities of all the *sip1-i4* mutant cells (Figure 3A, *sip1-i4* + *rho3*TN, *sip1-i4* + *rho3*EV).

Next, we assessed whether various forms of Rho3 are associated with Sip1. For this purpose, wild-type Rho3, the nucleotide-locked forms of Rho3 [GTPases in either the GTP-bound (Rho3G22V) or GDP-bound (Rho3T27N) confirmation] and a Rho3E48V effector domain mutant were fused to GFP and expressed using an inducible *nmt1* promoter. These cells were used to prepare lysates that were then used in binding experiments in which purified full-length Sip1 was fused to GST protein. The results showed that the Sip1-GST protein bound to each of the various forms of Rho3 (Figure 3B upper panel). GST protein did not associate with each Rho3 protein (Figure S1A). Quantification of the 3 independent experiments showed that Sip1 binding to Rho3EV was a little weaker than that to wild-type Rho3, and Sip1 bound to Rho3TN to almost the same degree as that to wild-type Rho3 (Figure 3B lower panel). Thus, binding strengths between Sip1 and these mutant forms of Rho3 versus wild-type Rho3 did not differ significantly as compared with the clear difference in the ability of each Rho3 mutant to rescue the *sip1-i4* mutant cells (Figure 3A). This was different from the binding between Apm1 and Rho3, which showed nucleotide -dependence and effector domain sensitivity [10]. Therefore, we hypothesized that although Sip1 could bind to Rho3, Sip1 may not serve as the effector of Rho3.

### Sip1 is required for Rho3 localization at the Golgi/endosomes

**Figure 4:**
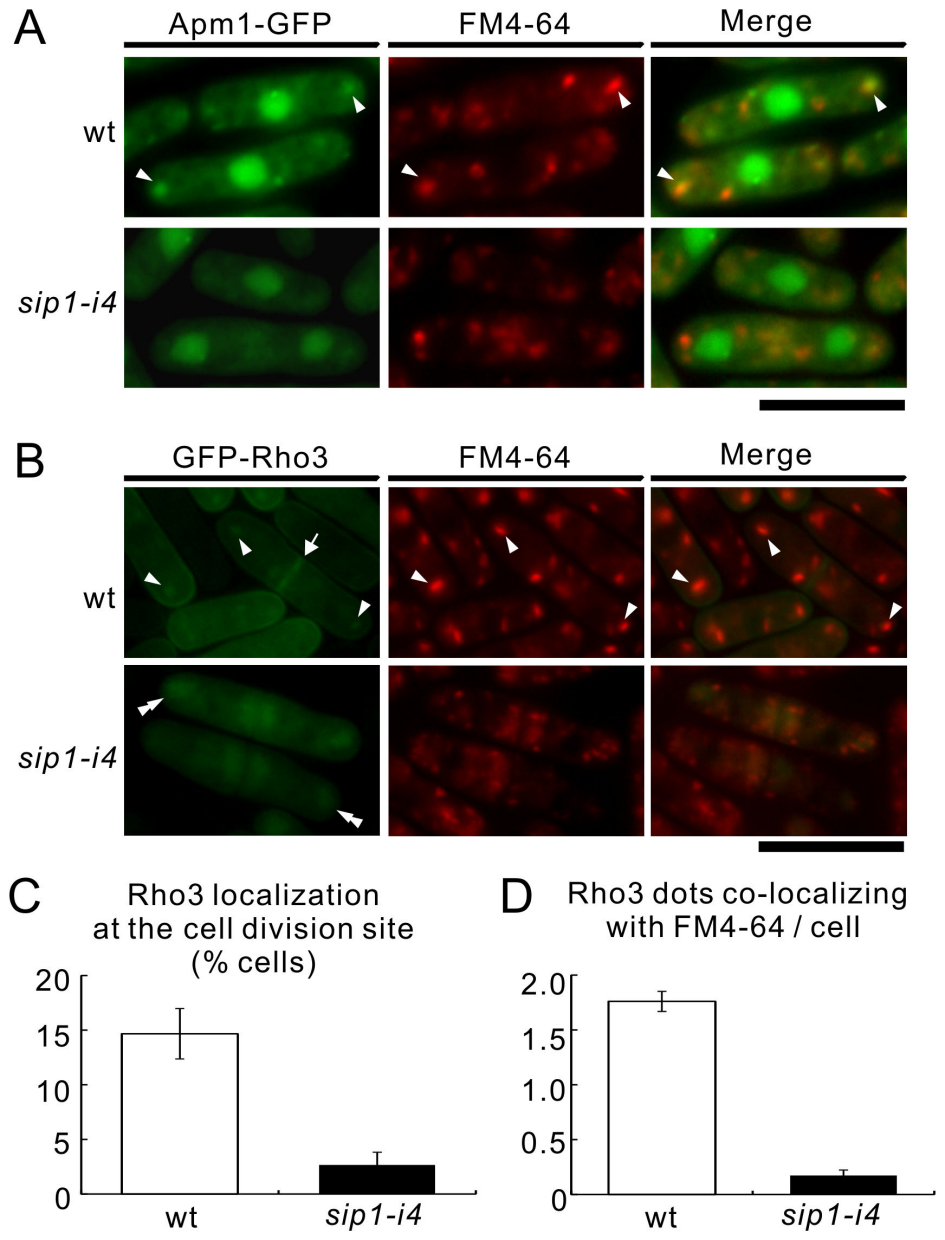
Both of Rho3 and Apm1 fail to co-localize at the Golgi/endosomes in *sip1-i4* mutant cells. (A) Subcellular localization of Apm1-GFP in wild-type (wt) and *sip1-i4* mutant cells (*sip1-i4*). Cells expressing Apm1-GFP were cultured in YPD medium at 27°C, were incubated with the dye FM4-64 for 5 min at 27°C to visualize the Golgi/endosomes. The fluorescence of the FM4-64 was examined under the fluorescence microscope. Arrowheads indicated the localization of Apm1-GFP to the Golgi/endosomes. Bar, 10 µm. (B) Subcellular localization of GFP-Rho3 in wild-type (wt) and *sip1-i4* mutant cells (*sip1-i4*). Cells expressing Rho3 were cultured in YPD medium at 27°C, following which they were incubated with FM4-64 dye for 5 min at 27°C to visualize the Golgi/endosomes. FM4-64 fluorescence was examined using a fluorescence microscope. Arrowheads indicate the dot-like structures of GFP-Rho3 and the Golgi/endosomes stained with FM4-64, double arrowheads indicate cytoplasmic accumulation, and arrows indicate the concentrated fluorescence at the cell division site. Bar, 10 µm. (C) Percentage of cells in which Rho3 were localized at the cell division site in wild-type (wt) and *sip1-i4* cells. (D) Quantitative analysis for the number of Rho3 dots co-localizing with FM4-64/cells in wt and *sip1-i4* cells. Cells in C and D were incubated as those in B. Data are the means ± standard deviations of 3 independent experiments with 150 cells in C and D.

We have previously demonstrated that the endosomal localization of the AP-1 complex, including Apm1 (Figure 4A, arrowheads) was nearly abolished in the *sip1-i4* mutant cells [13], demonstrating a conserved role of Sip1 in recruiting the AP-1 complex to the Golgi/endosomes. This led us to investigate the effect of the *sip1-i4* mutation on the intracellular localization of Rho3. For this, we used GFP-tagged Rho3 that was chromosomally expressed in the wild-type and *sip1-i4* mutant cells. In wild-type cells, GFP-Rho3 protein was localized in the Golgi/endosomes in addition to the plasma membrane and division site [10]. GFP-Rho3 fluorescence was observed as dot-like structures that co-localized with FM4-64-positive structures (Figure 4B arrowheads) as well as at the plasma membrane and division site (Figure 4B, arrows) in the wild-type cells. In contrast, in the *sip1-i4* mutant cells, the localization of GFP-tagged Rho3 to the division site was greatly impaired (Figure 4B, *sip1-i4*). In addition, in the *sip1-i4* mutant cells, Rho3 was observed as large clusters in the cytoplasm (Figure 4B, *sip1-i4*, double arrowheads) and the number of Rho3 dots that co-localized with FM4-64 was markedly lower compared with the wild-type cells (Figure 4B). The above findings were also supported by the quantification of Rho3 localization at the division site (Figure 4C) and Rho3 dots co-localization with FM4-64 (Figure 4D) in the wild-type and *sip1-i4* cells. We raised antibodies against *Schizosaccharomyces pombe* Rho3 protein and examined the expression level of endogenous Rho3 in wild-type and *sip1-i4* mutant cells. The immunoblotting data showed that the amount of Rho3 protein in *sip1-i4* mutant cells was slightly less, by 20%, than in wild-type cells (Figure S2). This raises the possibility that Rho3 protein may become unstable if it fails to localize in Golgi/endosomes.

We next examined the intracellular localization of endogenous Rho3 protein by performing immunostaining using the polyclonal- and monoclonal-Rho3 antibodies. However, numerous dot-like structures were observed both in wild-type and Rho3-deleted cells (Figure S3). Furthermore, the expected Rho3 localization to the plasma membrane was not observed in wild-type cells at the endogenous level (Figure S3). We, therefore, concluded that the Rho3 antibodies did not properly recognize endogenous Rho3 protein *in vivo* and that the numerous dots detected by the Rho3 antibodies may include somewhat artificial structures.

### The *Sip1-i4* mutant protein can interact with the AP-1 complex and Rho3

**Figure 5:**
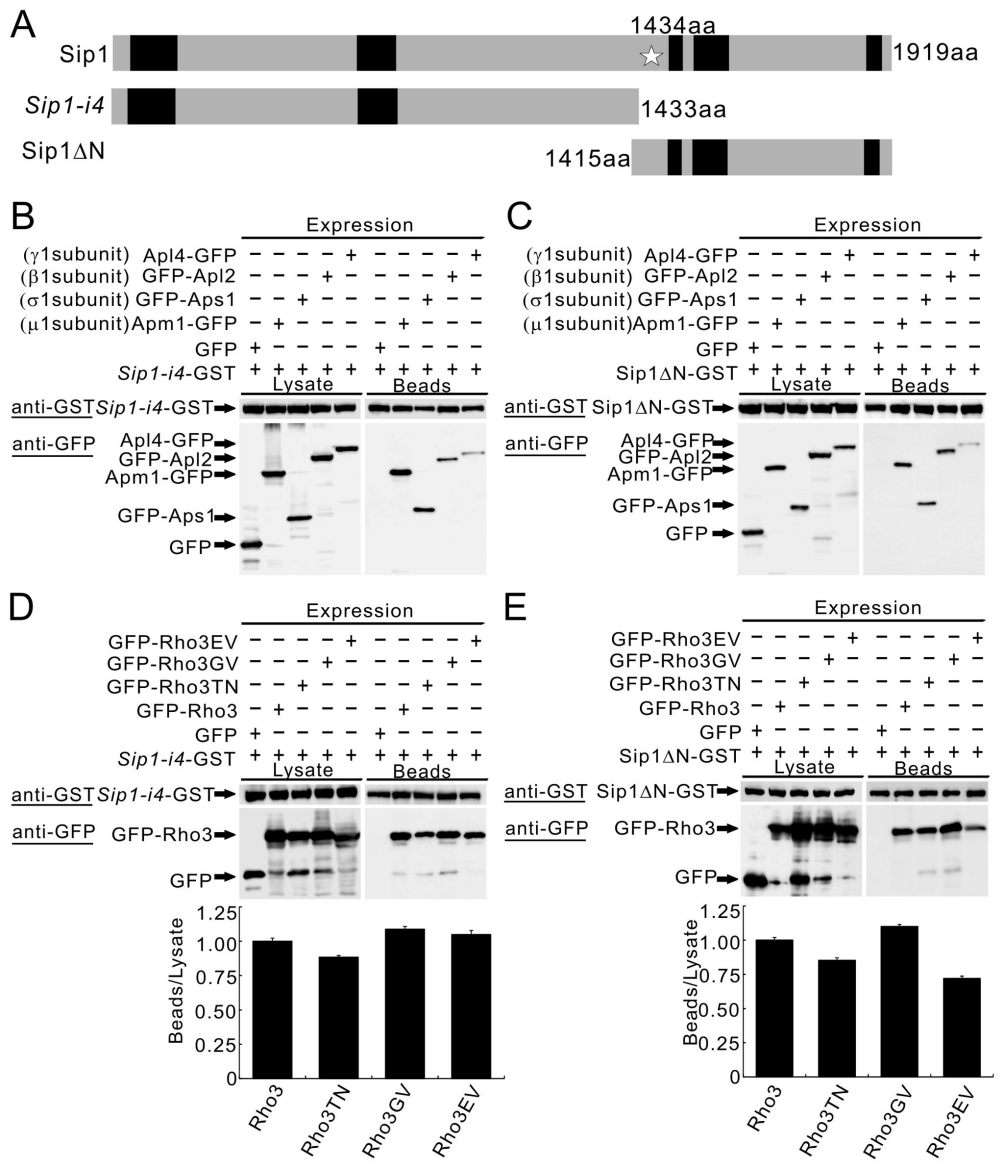
The Sip1 C-terminus is dispensable for the Sip1 association with the AP-1 complex or Rho3. (A) Schematic representation of Sip1 protein, *Sip1-i4* mutant protein and Sip1ΔN protein. The Sip1 protein is 1919 amino acids long and contains HEAT repeats (black). Star represents termination codon at the amino acid position 1434 found in the *sip1-i4* allele. The *Sip1-i4* mutant protein lacks the C-terminal 485 amino acids. The Sip1ΔN protein lacks the N-terminal 1414 amino acids. (B) Binding assay involving *Sip1-i4* and the 4 subunits of the AP-1 complex. GST pull-down experiments were performed using *Sip1-i4*-GST, *Sip1-i4*-GST expressed under the control of the *nmt1* promoter. Cells that expressed GFP alone or GFP-tagged to the 4 subunits of the AP-1 complex were harvested, and their lysates were incubated with the purified *Sip1-i4* fused GST protein. GST-tagged proteins were analyzed as shown in Figure 3B. (C) Binding assay involving Sip1ΔN and the 4 subunits of the AP-1 complex. The binding assay was performed as described in B. (D) Binding assay involving *Sip1-i4* and various mutant forms of Rho3. GST pull-down experiments were performed using *Sip1-i4*-GST expressed under the control of the *nmt1* promoter. Cells that expressed GFP alone or various GFP-tagged mutant forms of Rho3 were harvested, and their lysates were incubated with the purified *Sip1-i4-*GST protein. GST-tagged *Sip1-i4* was precipitated with glutathione beads, washed extensively, subjected to SDS-PAGE, and immunoblotted using anti-GFP or anti-GST antibodies. (E) Binding assay involving Sip1ΔN and various mutant forms of Rho3. The binding assay was performed as described in (D). Lower panel: Quantitation of GFP-tagged various mutant forms of Rho3 beads protein levels by densitometry of the expressed bands against that of the lysate protein levels as shown in D and E. Data from at least three independent experiments are expressed as means ± standard deviations.

In our previous study, we showed that Sip1 played a role in recruiting the AP-1 complex to the Golgi/endosomes through physical interaction, and here we showed that in the *sip1-i4* mutant cells both Rho3 and the AP-1 complex were mislocalized. Therefore, we examined the effect of the *sip1-i4* mutation on the physical interaction between Sip1/Rho3 and the Sip1/AP-1 complex. The *sip1-i4* mutation resulted in a truncated protein product that lacked 485 amino acids at the C-terminus of the Sip1 protein (Figure 5A, *Sip1-i4*). To monitor the interaction of AP-1 complex with the *Sip1-i4* mutant protein, we generated a GST-tagged mutant lacking the 485 amino acids at the C-terminus (*Sip1-i4*-GST). We examined whether *Sip1-i4* mutant protein associate with the AP-1 complex. For this purpose, purified *Sip1-i4*-GST protein was used in binding experiments with lysates prepared from the cells expressing Apm1, Apl2, Apl4, and Aps1 fused to GFP or a control GFP protein. These results showed that the C-terminally truncated *Sip1-i4* mutant protein bound to the AP-1 complex (Figure 5B). GST protein did not associate with the AP-1 complex (Figure S4). We also tested whether the binding between Sip1 and the AP-1 complex is dependent on the C-terminal region of Sip1. For this purpose, we expressed the C-terminal region of Sip1, lacking the 1414 amino acids at the N-terminus (Figure 5A Sip1ΔN), and performed the binding experiment using purified Sip1ΔN-GST (Figure 5C). The results showed that the C-terminal portion of the Sip1 protein bound to the AP-1 complex (Figure 5C).

Similar binding experiments were performed using the 2 truncated Sip1 mutant proteins fused to GST and various versions of GFP-fused Rho3 proteins or the control GFP. The results showed that various forms of Rho3 bound to both the truncated versions of the Sip1 protein (Figure 5D, E). Thus, either the N-terminal or the C-terminal part of Sip1 can bind to Rho3 and the AP-1 complex. As shown in Figure 5A, the 2 truncated Sip1 mutant proteins harbour multiple HEAT (Huntington-elongation-A subunit-TOR) interacting domains, the interaction between Sip1 and these proteins may be achieved through these domains. Therefore, we reasoned that the mislocalization of Rho3 in the *sip1-i4* mutant cells may not be derived from the loss of association between Rho3 and the *Sip1-i4* mutant proteins and may involve mechanism other than protein interaction.

### The C-terminus of Sip1 is important for its Golgi/endosomal localization

**Figure 6:**
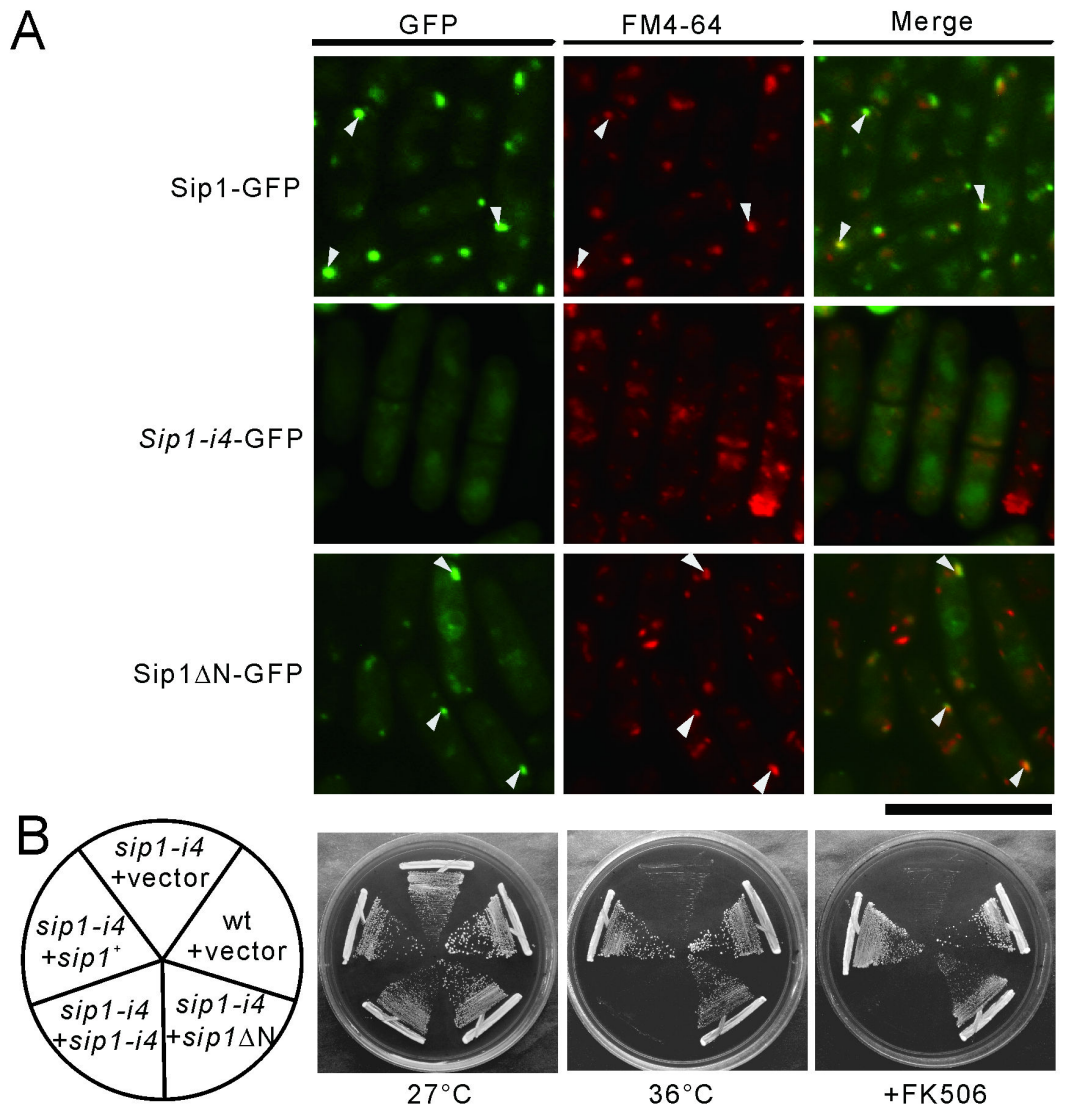
The C-terminus of Sip1 is important for its Golgi/endosomal localization. (A) Co-localization of Sip1-GFP, *Sip1-i4*-GFP or Sip1ΔN-GFP with FM4-64 in wild-type cells. The wild-type (wt) cells that expressed chromosome-borne Sip1-GFP or wt cells transformed with pREP1-*Sip1-i4*-GFP or pREP1-Sip1ΔN-GFP were examined by fluorescence microscopy under repressed conditions. The cells were incubated with FM4-64 fluorescent dye for 5 min at 27°C to visualize the Golgi/endosomes. FM4-64 fluorescence was examined using a fluorescence microscope. Arrowheads indicate the dot-like structures and the Golgi/endosomes. Bar, 10 µm. (B) Sip1ΔN suppresses *sip1-i4* cells similar to Sip1. The wt and *sip1-i4* cells were transformed with the pDB248 multi-copy vector or the vector containing *sip1* ^*+*^, *sip1-i4*, and *sip1*ΔN expressed under the control of the *nmt1* promoter. Cells were streaked onto plates containing 0.5 µg/mL FK506 and then incubated at 27°C for 4 d or at 36°C for 3 d, respectively.

To search for the mechanism by which Sip1 can recruit Rho3 at the Golgi/endosomes, we analyzed the effect of the *sip1-i4* mutation on its localization. For this purpose, we expressed the *Sip1-i4* mutant protein fused to GFP (*Sip1-i4*-GFP). Full-length Sip1 was localized to dot-like structures that mostly co-localized with FM4-64 (Figure 6A, Sip1-GFP arrowheads). In contrast, *Sip1-i4*-GFP failed to localize to the Golgi/endosomes because the specific dot-like structures were rarely observed (Figure 6A, *Sip1-i4*-GFP). Instead, they were diffusely localized in the cytoplasm.

To further investigate whether the C-terminal region of Sip1 that is deleted in the *Sip1*-*i4* mutant protein plays a critical role in its localization, we expressed Sip1ΔN as a GFP-fusion protein (Sip1ΔN-GFP). Notably, Sip1ΔN-GFP was localized in the dot-like structures similar to those of full-length Sip1-GFP (Figure 6A, Sip1ΔN-GFP). In addition, the Sip1ΔN-GFP dots co-localized with FM4-64-positive structures during an early stage of endocytosis (Figure 6A, Sip1ΔN-GFP, arrowheads). The amount of these 2 truncated Sip1 mutant proteins and the full-length Sip1 protein did not differ significantly (data not shown) indicating that the reduced level of localized signal of Sip1 in the *Sip1-i4*-GFP is not due to a decrease in overall Sip1 levels in the *Sip1-i4* mutant. These results suggest that the C-terminus of Sip1 is required and sufficient for its Golgi/endosomal localization.

We also assessed the ability of these truncated mutant Sip1 fragments to rescue the phenotypes of the *sip1-i4* mutant cells. The results revealed that the *sip1-i4* mutant fragment failed to suppress the phenotypes (Figure 6B, +*sip1-i4*), whereas the C-terminal region of Sip1 rescued the mutant phenotypes (Figure 6B, +*sip1*ΔN), including the sensitivity to heat and FK506. This indicated that there is a correlation between the suppression ability and correct localization of these truncated gene products.

### Sip1 links Rho3 to AP-1 complex

**Figure 7:**
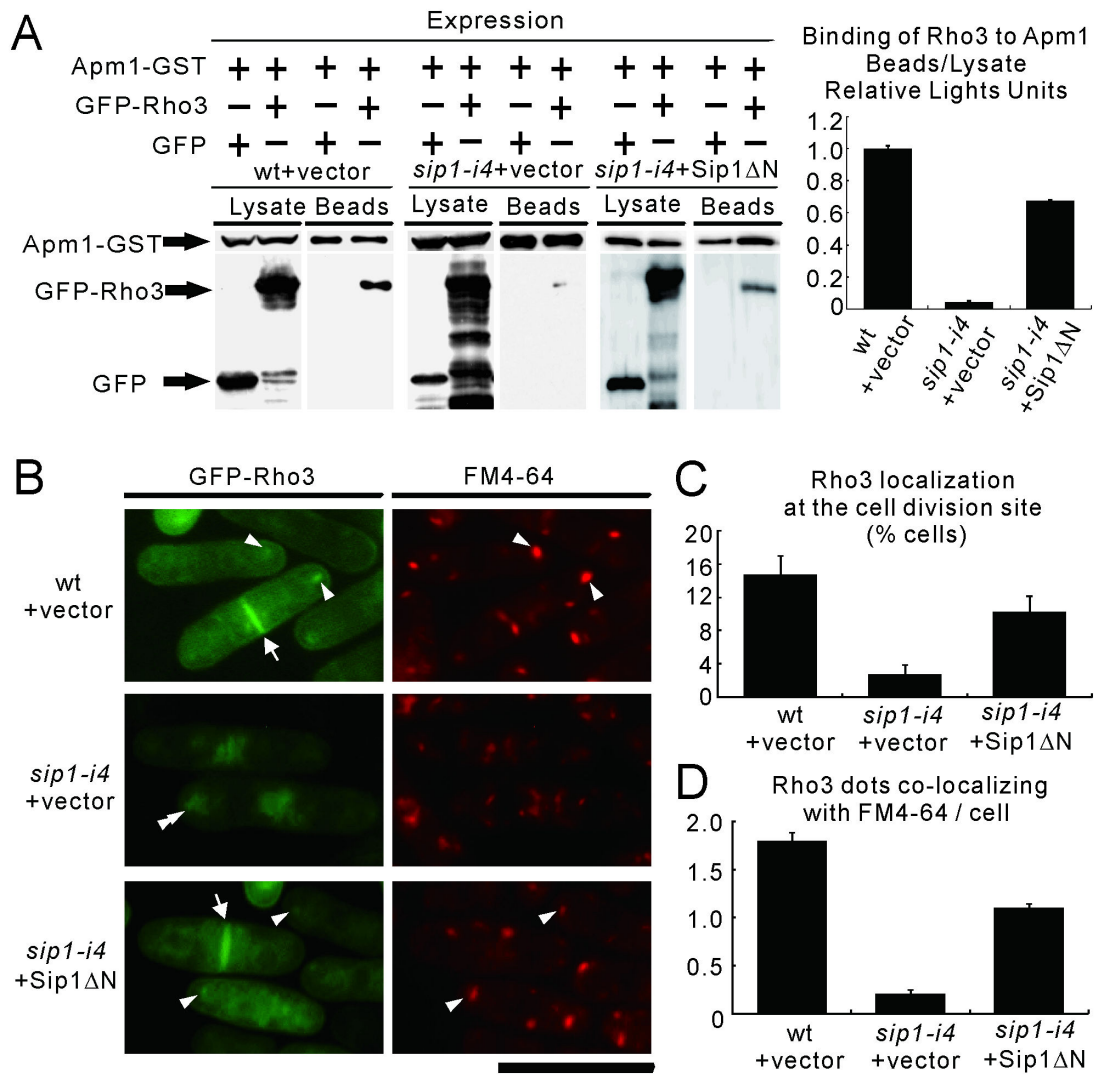
Sip1 links Rho3 to AP-1 complex. (A) Binding assay involving Apm1 and Rho3 in wild-type, or *sip1-i4* cells harboring the control vector or Sip1ΔN. GST pull-down experiments were performed using Apm1-GST expressed in wild-type (wt) and *sip1-i4* mutant (*sip1-i4*) cells, which were transformed with the pDB248 multi-copy vector or the vector containing *sip1*ΔN expressed under the control of the *nmt1* promoter. Cells that expressed GFP alone or GFP-Rho3 were harvested, and their lysates were incubated with purified full-length Apm1 fused to GST. Proteins bound to glutathione beads were analyzed by SDS-PAGE and visualized by autoradiography. Right panel: Quantitation of GFP-Rho3 beads protein levels by densitometry of the expressed bands against that of the lysate protein levels in wild-type cells, *sip1-i4* cells or *sip1-i4* cells with Sip1ΔN expression as shown in A. Data from at least three independent experiments are expressed as means ± standard deviations. (B) Subcellular localization of GFP-Rho3 in wild-type cells, *sip1-i4* cells or *sip1-i4* cells with Sip1ΔN expression. GFP-Rho3 expressed in wild-type (wt) and *sip1-i4* mutant (*sip1-i4*) cells, which were transformed with the pDB248 multi-copy vector or the vector containing *sip1*ΔN expressed under the control of the *nmt1* promoter. Cells were cultured in YPD medium at 27°C, following which they were incubated with FM4-64 dye for 5 min at 27°C to visualize the Golgi/endosomes. FM4-64 fluorescence was examined using a fluorescence microscope. Arrowheads indicate the dot-like structures of GFP-Rho3 and the Golgi/endosomes stained with FM4-64, double arrowheads indicate cytoplasmic accumulation of GFP-Rho3, and arrows indicate the concentrated fluorescence at the cell division site. Bar, 10 µm. (C) Percentage of cells in which Rho3 were localized at the cell division site in wild-type (wt) and *sip1-i4* cells, which were transformed with the pDB248 multi-copy vector or the vector containing *sip1*ΔN expressed under the control of the *nmt1* promoter. (D) Quantitative analysis for the number of Rho3 dots co-localizing with FM4-64/cells in wt and *sip1-i4* cells, which were transformed with the pDB248 multi-copy vector or the vector containing *sip1*ΔN expressed under the control of the *nmt1* promoter. Data are the means ± standard deviations of 3 independent experiments with 150 cells in B.

Because the *sip1-i4* mutation affected the localization of both the AP-1 complex and Rho3 protein to the Golgi/endosomes and our previous findings showed that Rho3 formed a complex with Apm1 in the Golgi/endosomes [10], we investigated whether Sip1 is required for the association of between Apm1 and Rho3. Therefore, we examined the binding of Apm1-GST and GFP-Rho3 in wild-type and *sip1-i4* mutant cells. The results showed that the binding of Apm1 to Rho3 in the *sip1-i4* mutant cells was greatly impaired as compared in wild-type cells (Figure 7A), suggesting that Sip1 is required for the physical interaction between Apm1 and Rho3.

It should be noted that many bands with smaller molecular weights than that of the full-length GFP-fused Rho3 protein were detected by SDS-PAGE in the *sip1-i4* mutant cells (Figure 7A). We hypothesized that Rho3 protein may become unstable when it fails to be localized to the Golgi/endosomal membranes. In support of this possibility, immunoblotting data using antibodies raised against Rho3 protein showed that the amount of Rho3 protein in *sip1-i4* mutant cells was slightly less, by 20%, than that in wild-type cells (Figure S2).

We also examined whether the interaction between Apm1 and Rho3 could be rescued in *sip1-i4* cells by Sip1ΔN expression. The results showed that the expression of Sip1ΔN significantly rescued the binding between Apm1 and Rho3 (Figure 7). Furthermore, we examined the effect of Sip1ΔN expression on the intracellular localization of Rho3 and its co-localization with FM4-64. Notably, Sip1ΔN expression also rescued Rho3 co-localization with FM4-64, namely, Golgi/endosome localization (arrowheads) as well as localization to the septa (arrows) (Figure 7B–D).

We also performed IP experiments shown in Figure 3B Figures 5B, 5C, 5D, 5E, and Figure 7A, using GFP-fused Rho2, and demonstrated that Rho2 protein did not interact with the GST-fused full-length Sip1, Sip1-i4, Sip1ΔN or Apm1 protein, thus confirming the specificity of the interactions detected in each experiment (Figure S5). 

## Discussion

In this study, we present a novel role for Sip1, a conserved AP-1 accessory protein, in recruiting the Rho3 small GTPase to the Golgi/endosomes, on the basis of our discovery of the functional and physical interactions between Rho3 and Sip1. It has been established that Sip1 recruited the AP-1 complex to the Golgi/endosomes physical interaction [13] and that Rho3 is involved in the regulation of the Golgi/endosomal trafficking by functionally and physically interacting with Apm1 [10]. The findings that Sip1 regulates proper localization of Rho3 and the AP-1 complex and that Sip1 is associated with Rho3 and the AP-1 complex suggested that Sip1 links Rho3 signaling to AP-1 complex-mediated Golgi/endosomal trafficking.

Sip1 is highly conserved throughout evolution, with homologs from human to yeast. A previous study in *S. cerevisiae* and humans also demonstrated the role of the AP-1 accessory proteins Laa1 (*la*rge AP-1 accessory) and p200 [15] in AP-1-mediated transport, and Laa1 is involved in AP-1 localization to the trans-Golgi network (TGN) [14]. Interestingly, aftiphilin, another AP-1 interacting protein, co-elutes with two other AP-1 binding partners, p200a and γ-synergin [20], and it has been suggested that the aftiphilin/p200/γ-synergin complex may have additional functions along with its role in facilitating AP-1 function [15]. Notably, there are differences in the phenotypes associated with the loss of function of the AP-1 accessory protein in both yeasts. The *sip1* ^+^ gene is essential for growth, whereas the budding yeast *laa1* null cells are viable and exhibited synthetic growth defects when combined with *gga1*Δ*gga2*Δ [14]. Furthermore, the temperature-sensitive *sip1-i4* mutants displayed distinct phenotypes ranging from defects in Golgi/endosomal trafficking and vacuole fusion [13] to cytokinesis defects [21] even at the permissive temperature, whereas *laa1* deletion alone did not impair the secretion of mature α-factor and transport of CPY and showed synthetic effects when combined with *gga1gga2* double deletion [14]. The reason for the differences could be that *S. pombe* Sip1 might be required for the proper function of protein(s) other than AP-1. Our phenotypic screening using the temperature-sensitive growth defect of the *sip1-i4* allele was successful in identifying *rho3* ^+^ as a multi-copy suppressor of the *sip1* mutant and revealed an additional functional interaction between the AP-1 accessory protein and Rho3.

How overexpression of *rho3* ^*+*^ can suppress the phenotypes of *sip1-i4* cells even in the absence of clear Golgi/endosome localization remains unclear. However, Rho3 overproduction suppressed the *sip1-i4* mutant phenotypes, indicating that Rho3 exerts its effects in the absence of its clear Golgi/endosome localization. Rho3 was previously isolated as a multi-copy suppressor of several mutant alleles of a component of the exocyst complex including Sec4 and Sro7 in budding yeast and Sec8 in fission yeast [22]. The exocyst complex is highly conserved from yeasts to mammals and is involved in the late stages of exocytosis by targeting and tethering post-Golgi vesicles to the plasma membrane. Thus, Rho3 overproduction may stimulate secretion via the components of the exocyst by locally increasing the activity of the exocytic apparatus, which leads to the suppression of mutant strains with defective exocytosis including *sip1-i4* mutant cells. However, we prefer the alternate possibility that, even though there is no clearly visible Rho3 protein co-localizing with FM4-64 in the *sip1-i4* mutant cells, an extremely small amount of Rho3 protein may still exist in Golgi/endosomes, which can be augmented by the overproduction of Rho3 and result in the suppression of the *sip1-i4* mutant phenotypes. In support of this possibility, the forced overexpression of GFP-Rho3, by culturing the cells in the absence of thiamine (induced condition), visualized some Rho3 dots co-localizing with FM4-64 on the *sip1-i4* mutant background (data not shown).

Our previous reports revealed that Rho3 associates with the AP-1 complex in a GTP and effector-dependent manner [10], whereas the association of Sip1 with Rho3 appears to be GTP-independent. Notably, several studies in higher eukaryotes reported that Rac can directly interact with PIP5K isoforms in a GTP-independent manner [23–25], and unlike the interaction of Rac with most other effectors, the interaction between Rac and PIP5K requires the C-terminal polybasic region of Rac. We then investigated the localization dependency between Rho3/Sip1. Reciprocal experiments illustrated that Sip1-GFP co-localization with FM4-64 (Figure S6A, arrowheads), and with the trans-Golgi protein Sec72-mCherry (Figure S6B, arrowheads), were observed in Δ*rho3* cells similar to those observed in wild-type cells. Thus, although the *sip1-i4* mutation affects Rho3 localization to the division site and the Golgi/endosomes, Rho3 deletion did not affect Sip1 localization to the Golgi/endosomes. These data are consistent with the proposed role of Sip1 as a regulator, but not as an effector of Rho3.

If Sip1 is providing a physical link between Rho3 and AP-1 complex and the interaction with Rho3 is nucleotide independent, then how the Rho3/AP-1 interaction would be dependent on the GTP-bound form of Rho3? We then examined and demonstrated the importance of the C-terminal region of Sip1 for its intracellular localization. The *sip1-i4* mutation resulted in a termination codon at amino acid position 1434 located within the highly conserved region (HCR), which contains an approximately 200-amino acid segment conserved throughout evolution in this protein family [14]. Notably, the Sip1 C-terminal region (Sip1ΔN) was sufficient for its Golgi/endosomal localization, suppression for the *sip1-i4* mutant cells, and for the association of Sip1 with Rho3 and the AP-1 complex (Figures 5, 6). In addition, although the *Sip1-i4* mutant protein that failed to localize in the Golgi/endosome (Figure 6A) maintained its ability to bind to Rho3 and AP-1, the *Sip1-i4* mutant protein lost its ability to suppress the *sip1-i4* mutant cells (Figures 5, 6). Thus, we hypothesize that Sip1 can bind to Rho3 and the AP-1 complex in the cytosol and recruit them to the Golgi/endosomes, thereby enhancing the formation of the Rho3/AP-1 complex, which is dependent on the GTP-bound form of Rho3. Consistently, *rho3* ^*+*^ overexpression suppressed the phenotypes of the *sip1-i4* mutant cells in a GTP- and effector-dependent manner, as Rho3TN and Rho3EV failed to suppress the *sip1-i4* mutant cells, even though the binding between Rho3 and Sip1 was GTP-independent. Because our previous findings indicated that *rho3* ^*+*^ overexpression can suppress *apm1-1* mutant cells and that Rho3/AP-1 binding was GTP-dependent [10], the suppression of the *sip1* mutant phenotypes by Rho3 overproduction may reflect the increase in the amount of the Rho3/AP-1 complex in the Golgi/endosomes. Sip1 possesses 5 HEAT (Huntington-elongation-A subunit-TOR) repeat domains implicated in protein–protein interactions, and 2 of the HEAT repeat domains localize in the C-terminal region [13]. Therefore, the interaction of Sip1 with unidentified protein(s) through HEAT repeat domains in the C-terminus may direct Sip1 to the Golgi/endosomes.

The prenylation of small GTPases including Rho, is a well-known mechanism for targeting the Rho family proteins to the membrane and their proper cellular location. However, specific targeting factors for each small GTPase have not been completely characterized. In the present study, we presented a novel role of Sip1 in Rho localization to specific membranes, and given the high conservation of the AP-1 accessory protein and small GTPases, our discovery may shed light on the understanding of the regulatory mechanisms of the membrane trafficking system mediated by Rho and the clathrin adaptor complex.

## Supporting Information

Figure S1 Binding assay involving GST and various mutant forms of Rho3. GST pull-down experiment was performed using GST expressed under the control of the *nmt1* promoter. Cells that expressed GFP alone or various GFP-tagged mutant forms of Rho3 were harvested, and their lysates were incubated with the purified GST protein. GST was precipitated with glutathione beads, washed extensively, subjected to SDS-PAGE, and immunoblotted using anti-GFP or anti-GST antibodies.
(TIF)

Figure S2 Expression level of endogenous Rho3 in wild-type cells and *sip1-i4* mutant cells. (A) Immunoblot analysis of the Rho3 protein in Rho3-deletion (Δ*rho3*), wild-type (wt) and *sip1-i4* mutant (*sip1-i4*) cells. The whole-cell lysates were analyzed by immunoblotting with polyclonal anti-Rho3 antibodies. (B) Quantitation of Rho3 protein levels by densitometry of the expressed bands against that of the tubulin protein levels in wild-type and *sip1-i4* cells shown in A.
(TIF)

Figure S3 Immunofluorescent Localization of Rho3 in wild-type and **Rho3-deletion cells**. The wild-type (wt) and Rho3-deletion cells (Δ*rho3*) were cultured in YES medium at 27°C. Cells were fixed and stained with ployclonal rat anti-Rho3 antibodies (A) and monoclonal rat anti-Rho3 antibody (B), and examined by fluorescence microscopy. Bar, 10 µm.
(TIF)

Figure S4 Binding assay involving GST and the 4 subunits of the AP-1 complex. GST pull-down experiment was performed using GST, expressed under the control of the *nmt1* promoter. Cells that expressed GFP alone or GFP-tagged to the 4 subunits of the AP-1 complex were harvested, and their lysates were incubated with the purified GST protein. GST was precipitated with glutathione beads, washed extensively, subjected to SDS-PAGE, and immunoblotted using anti-GFP or anti-GST antibodies.
(TIF)

Figure S5 Binding assay involving GFP-Rho2 and various GST fusion proteins. GST pull-down experiment was performed using GST-Sip1, *Sip1-i4*-GST, Sip1ΔN-GST and Apm1-GST, expressed under the control of the *nmt1* promoter. Cells that expressed GFP-Rho2 alone were harvested, and their lysates were incubated with the purified various GST fusion proteins. GST-fused proteins were precipitated with glutathione beads, washed extensively, subjected to SDS-PAGE, and immunoblotted using anti-GFP or anti-GST antibodies.
(TIF)

Figure S6 Subcellular localizations of Sip1-GFP in Rho3-deletion cells are similar to that in wild-type cells. (A) Subcellular localizations of Sip1-GFP in wild-type (wt) and Rho3-deletion cells (Δ*rho3*). Cells that expressed chromosome-borne Sip1-GFP were cultured in YPD medium at 27°C. They were incubated with FM4-64 dye for 5 min at 27°C to visualize Golgi/endosomes. Arrowheads indicate the localization of Sip1-GFP at Golgi/endosomes. Bar, 10 µm. (B) Sip1-GFP partially co-localized with the trans-Golgi marker Sec72-mCherry, but did not co-localize with the *cis*-Golgi marker Anp1-mCherry in Rho3-deletion cells (Δ*rho3*). Rho3-deletion cells expressed chromosome-borne Anp1-mCherry and Sip1-GFP, or chromosome-borne Sec72-mCherry and Sip1-GFP. Cells were cultured in YPD medium at 27^o^C and examined by fluorescence microscopy. Arrowheads indicate the co-localization of Sip1-GFP with Sec72-mCherry at trans-Golgi. Bar, 10 µm.
(TIF)
