# Supplementary figures and images for: Sip1, an AP-1 Accessory Protein in Fission Yeast, Is Required for Localization of Rho3 GTPase
Source: PLoS One. 2013 Jul 1;8(7):e68488. doi: 10.1371/journal.pone.0068488 (PMC3698097; doi:10.1371/journal.pone.0068488)

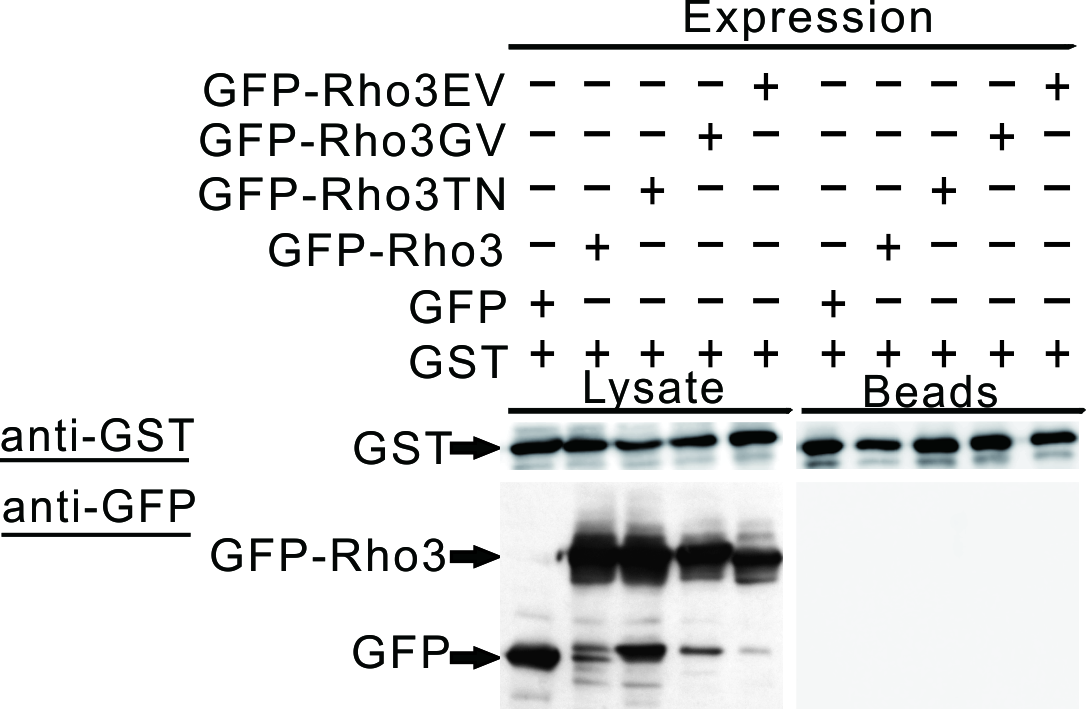

Supplement: Figure S1 — GST pull-down experiment was performed using GST expressed under the control of the nmt1 promoter. Cells that expressed GFP alone or various GFP-tagged mutant forms of Rho3 were harvested, and their lysates were incubated with the purified GST protein. GST was precipitated with glutathione beads, washed extensively, subjected to SDS-PAGE, and immunoblotted using anti-GFP or anti-GST antibodies. (TIF) [file pone.0068488.s001.tif]

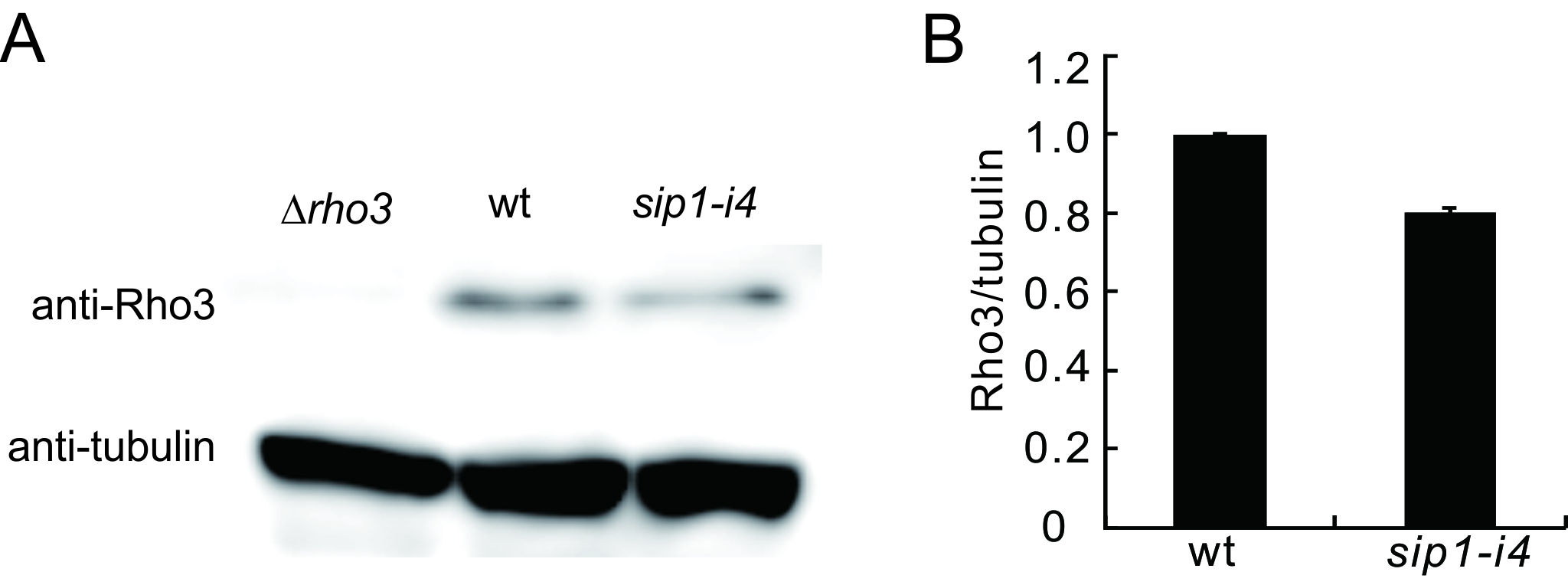

Supplement: Figure S2 — (A) Immunoblot analysis of the Rho3 protein in Rho3-deletion (Δrho3), wild-type (wt) and sip1-i4 mutant (sip1-i4) cells. The whole-cell lysates were analyzed by immunoblotting with polyclonal anti-Rho3 antibodies. (B) Quantitation of Rho3 protein levels by densitometry of the expressed bands against that of the tubulin protein levels in wild-type and sip1-i4 cells shown in A. (TIF) [file pone.0068488.s002.tif]

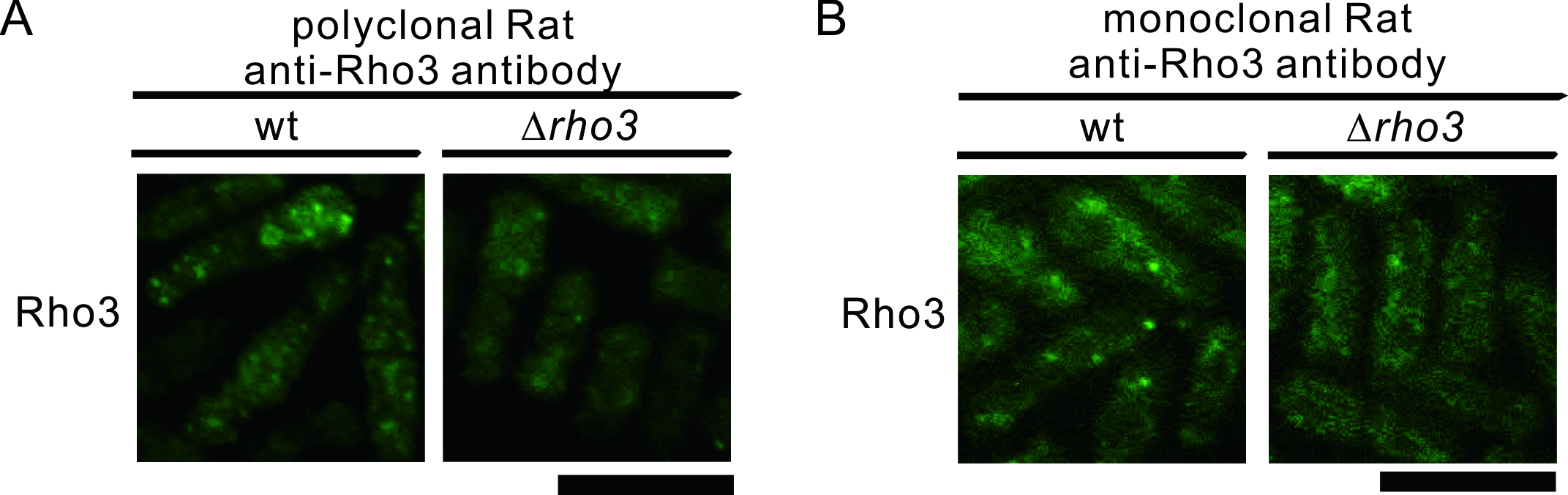

Supplement: Figure S3 — Rho3-deletion cells. The wild-type (wt) and Rho3-deletion cells (Δrho3) were cultured in YES medium at 27°C. Cells were fixed and stained with ployclonal rat anti-Rho3 antibodies (A) and monoclonal rat anti-Rho3 antibody (B), and examined by fluorescence microscopy. Bar, 10 µm. (TIF) [file pone.0068488.s003.tif]

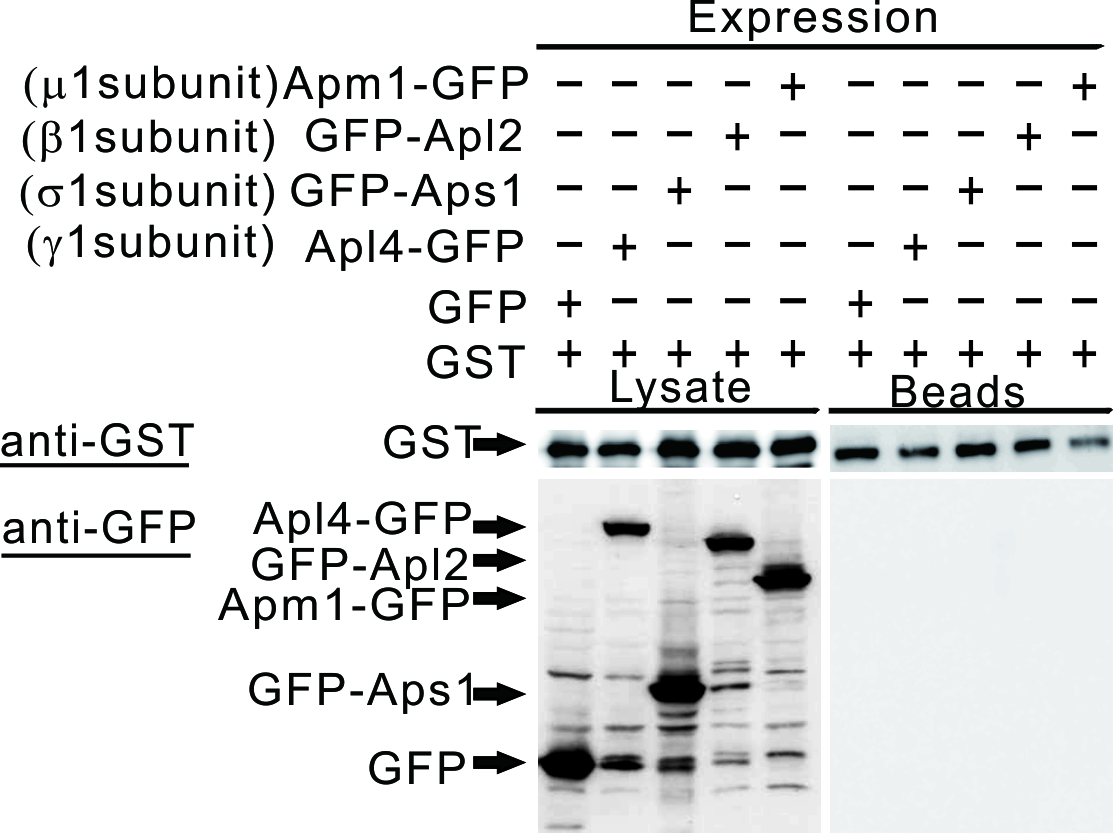

Supplement: Figure S4 — GST pull-down experiment was performed using GST, expressed under the control of the nmt1 promoter. Cells that expressed GFP alone or GFP-tagged to the 4 subunits of the AP-1 complex were harvested, and their lysates were incubated with the purified GST protein. GST was precipitated with glutathione beads, washed extensively, subjected to SDS-PAGE, and immunoblotted using anti-GFP or anti-GST antibodies. (TIF) [file pone.0068488.s004.tif]

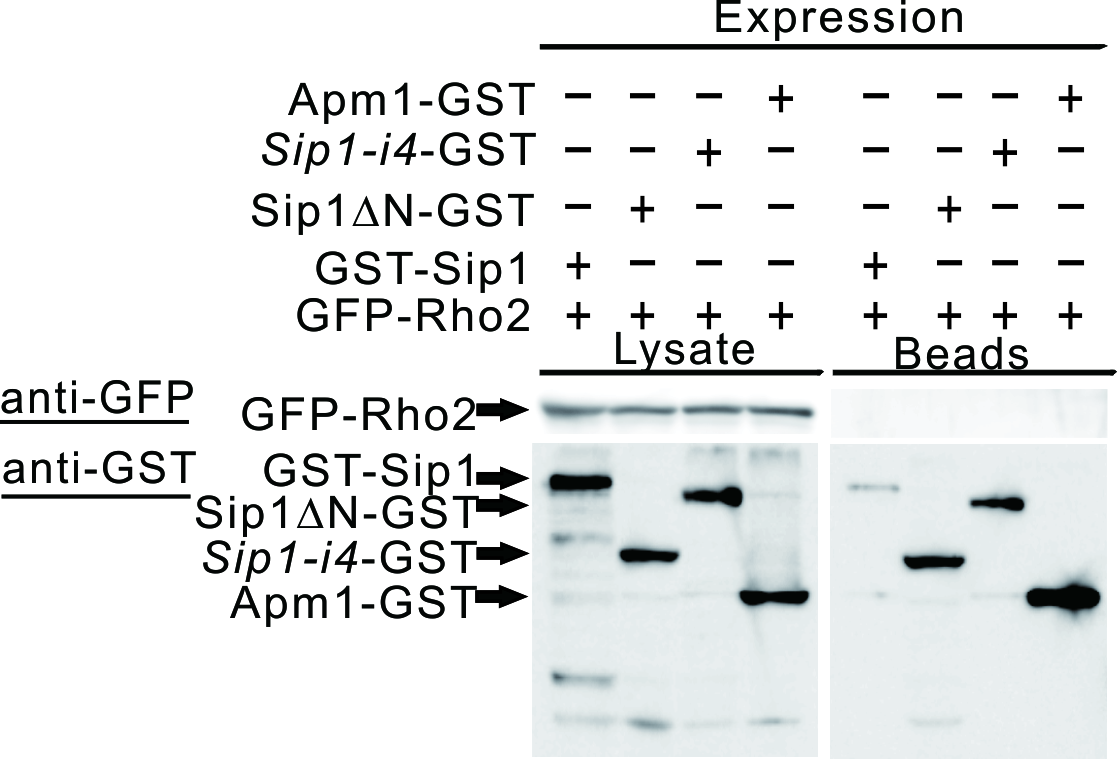

Supplement: Figure S5 — GST pull-down experiment was performed using GST-Sip1, Sip1-i4-GST, Sip1ΔN-GST and Apm1-GST, expressed under the control of the nmt1 promoter. Cells that expressed GFP-Rho2 alone were harvested, and their lysates were incubated with the purified various GST fusion proteins. GST-fused proteins were precipitated with glutathione beads, washed extensively, subjected to SDS-PAGE, and immunoblotted using anti-GFP or anti-GST antibodies. (TIF) [file pone.0068488.s005.tif]

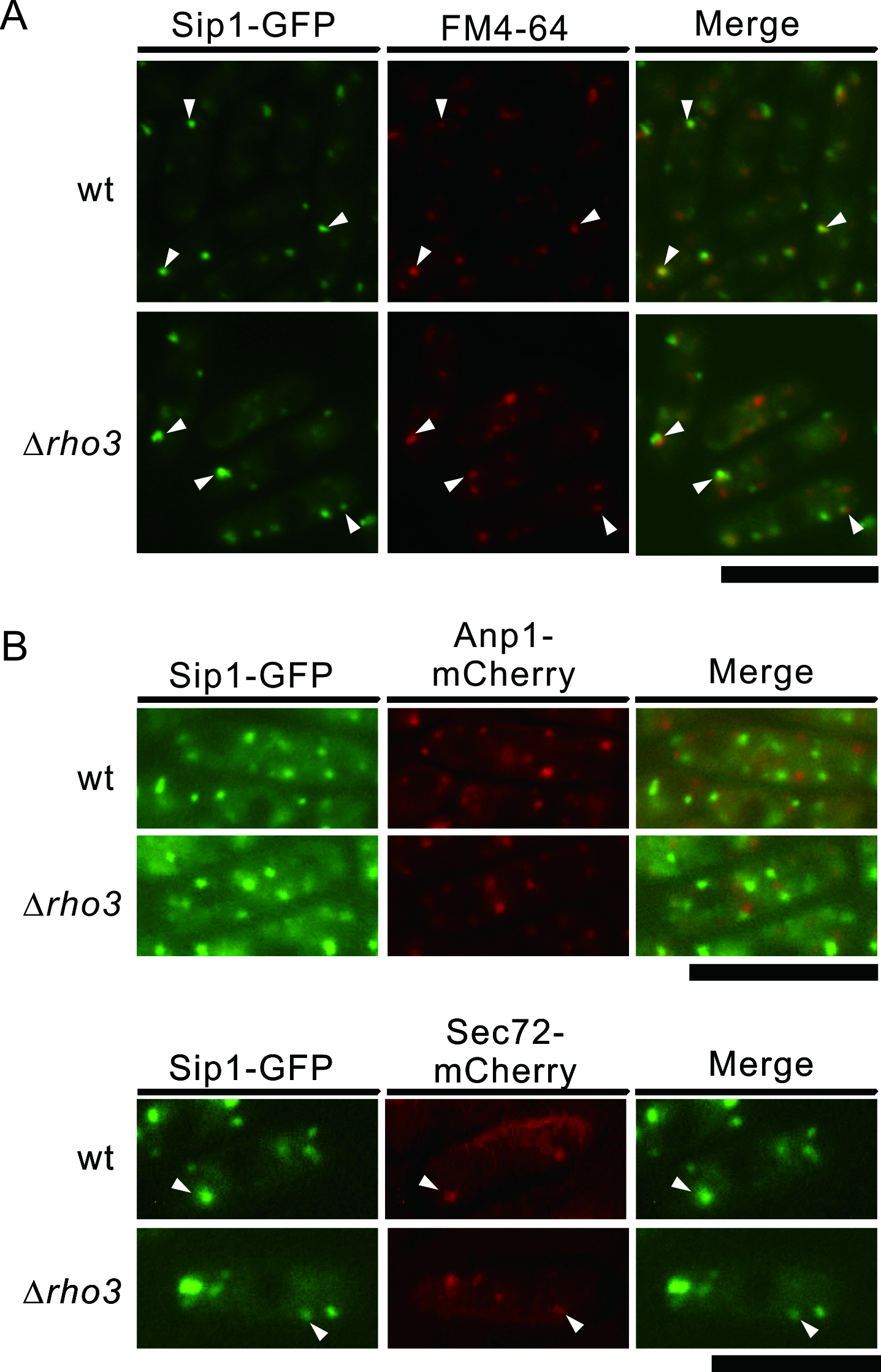

Supplement: Figure S6 — (A) Subcellular localizations of Sip1-GFP in wild-type (wt) and Rho3-deletion cells (Δrho3). Cells that expressed chromosome-borne Sip1-GFP were cultured in YPD medium at 27°C. They were incubated with FM4-64 dye for 5 min at 27°C to visualize Golgi/endosomes. Arrowheads indicate the localization of Sip1-GFP at Golgi/endosomes. Bar, 10 µm. (B) Sip1-GFP partially co-localized with the trans-Golgi marker Sec72-mCherry, but did not co-localize with the cis-Golgi marker Anp1-mCherry in Rho3-deletion cells (Δrho3). Rho3-deletion cells expressed chromosome-borne Anp1-mCherry and Sip1-GFP, or chromosome-borne Sec72-mCherry and Sip1-GFP. Cells were cultured in YPD medium at 27oC and examined by fluorescence microscopy. Arrowheads indicate the co-localization of Sip1-GFP with Sec72-mCherry at trans-Golgi. Bar, 10 µm. (TIF) [file pone.0068488.s006.tif]
